# Damage to *Sorubim cuspicaudus* Sperm Cryopreserved with Ethylene Glycol

**DOI:** 10.3390/ani13020235

**Published:** 2023-01-09

**Authors:** Víctor Atencio-García, Denia Padilla-Izquierdo, Juana Robles-González, Martha Prieto-Guevara, Sandra Pardo-Carrasco, José Espinosa-Araujo

**Affiliations:** 1 CINPIC, Fishculture Research Institute, School of Veterinary Medicine and Zootechnics, Department of Aquaculture Sciences, University of Córdoba, Montería 230002, Colombia; 2 School of Basic Sciences, Department of Biology, University of Córdoba, Montería 230002, Colombia; 3 School of Basic Sciences, Department of Mathematics and Statistics, Universidad de Córdoba, Montería 230002, Colombia; 4 School of Agricultural Sciences, Department of Animal Husbandry, National University of Colombia, Medellín 050034, Colombia

**Keywords:** cryodamage, plasma membrane, mitochondria, DNA fragmentation, reproduction

## Abstract

**Simple Summary:**

The study aimed to evaluate the damage suffered by cryopreserved semen of *S cuspicaudus* in mitochondria (Mit-d), plasmatic membranes (Mem-d), and DNA fragmentation (DNA-f) with an extender composed of ethylene glycol (6, 8, 10% EG), glucose (6%) and skim milk powder (5%). Sperm kinetics and damage in fresh, pre-frozen, and thawed semen were evaluated. In pre-frozen semen, sperm kinetics decreased, and Mit-d (11 to 13 times), Mem-d (2.4 to 4 times), and DNA-f (2.8 to 4 times) damages increased regarding fresh semen. In thawed semen, damage continued to rise in Mit-d (34 to 37 times), Mem-d (24.5 to 26.6 times), and DNA-f (13 to 18.5 times). Fertility (37–59%) and hatching (29–46%) rates of thawed-cryopreserved semen were reduced to half that recorded for fresh semen. In conclusion, the present study demonstrated that mitochondria, membrane, and DNA suffer considerable damage in pre-freezing and freezing–thawing, affecting their fertilizing capacity, which is reduced to half regarding fresh semen.

**Abstract:**

The study aimed to evaluate cryo-injury during the cryopreservation in *Sorubim cuspicaudus* sperm with ethylene glycol (EG) at different rates (6, 8, 10%). Fresh, prefrozen, and post-thawed sperm quality as motility total, velocities, mitochondria damage (Mit-d), membrane damage (Mem-d), and DNA fragmentation (DNA-f), were examined. The Mit-d, Mem-d, and DNA-f were evaluated through flow cytometry. High motility (>95%) and a low percentage of Mem-d (1.0 ± 0.5%), Mit-d (1.4 ± 0.9%), and DNA-f (2.4 ± 0.8%) were recorded for fresh semen. Prefrozen semen increases in Mit-d and DNA-f were observed compared to fresh semen (*p* < 0.05). In thawed semen, increased Mit-d (2.6 to 3-fold), Mem-d (6 to 1-fold), and DNA-f (3.3 to 6.6-fold) compared to prefrozen was observed. Thawed semen showed Mit-d (34 to 37-fold), Mem-d (24.5 to 26.6-fold) and DNA-f (13 to 18.5-fold) increased high. In conclusion, the present study demonstrated that mitochondria, membrane, and DNA integrity undergo significant damage during both pre-freezing and freezing/thawing with EG inclusion percentages from 6 to 10% that affect its fertilizing capacity, which is reduced to half of that obtained with fresh semen. It is suggested that a cryoprotective solution composed of 6% EG, 6% glucose, and 5% skimmed milk powder is a useful protocol for the cryopreservation of *S. cuspicaudus* semen.

## 1. Introduction

The shovelnose white catfish *Sorubim cuspicaudus* is a Neotropical catfish belonging to the Pimelodidae distributed in the Magdalena-Cauca and Sinú basins in Colombia and the lake basin Maracaibo in Venezuela [1]. This catfish is one of eighty-one freshwater species fishes reported in Colombia’s extinction vulnerable species list due to the intense fishing pressure and habitats destroyed [2]. It also has been considered a potential species for diversifying Colombia’s continental fish culture [3]. Low semen volume and reproductive asynchrony are the most frequent problems observed in hatchery farms in this catfish [4]. For this reason, the cryopreservation of their semen has been worked on with cryoprotectants such as dimethylacetamide (DMA) and ethylene glycol (EG) [3,4].

Sperm cryopreservation is a safe method for storing and preserving genetic material [5,6] and a conservation strategy for biodiversity programs for threatened or endangered species [7,8]. Moreover, it is a powerful approach in fish hatchery because it is a useful technique for effective and easy broodstock management and artificial fertilization [9]. However, injury effects of cryopreservation on sperm viability have been reported in many fish species as it causes damage to sperm cells produced by thermal shock, ice formation, and toxic and osmotic stress in the pre-freezing, freezing, and thawing phases [10,11,12,13]. Damage to the plasma membrane, mitochondria, DNA, and the morphology of cryopreserved sperm cells have been reported, affecting sperm viability and, ultimately, the fertilizing capability [5,13,14,15,16,17,18].

The plasma membrane is the first affected structure during cryopreservation due to chemical composition changes or environmental temperature. It is suggested that spermatozoa’s resistance to the freezing process and media toxicity is influenced by structural membrane lipid and protein chemical composition [11,19]. The plasma membrane is a major structure of motility activation [20]. Mitochondrial integrity and morphology are also considered critical factors for functional spermatozoa. Their damage decreases cell motility and ATP level after freezing and thawing, where oxidative stress plays a significant role in mitochondrial damage [21,22,23]. Sperm DNA integrity is associated with fertilization success and normal embryo or offspring development [18,24,25]. DNA integrity is an essential parameter in predicting the failure or success fertilizing capacity of semen [26,27,28] and the cryopreservation process. Oxidative stress has been suggested as one of the mechanisms responsible for DNA fragmentation due to the oxidation of specific bases, which may occur during the cryopreservation process [18,29,30,31]. There are few studies on spermatozoa injuries through the cryopreservation process of Neotropical fish.

Studies that examine semen damage in the cryopreservation process of Neotropical fish are scarce, but the works on *Prochilodus magdalenae* [15] and *Astrolebias minuano* [32] stand out. No reports are available in the literature on cryodamage to *S. cuspicaudus* sperm during cryopreservation. Therefore, this study aimed to evaluate the injuries suffered by *S. cuspicaudus* spermatozoa at the DNA, mitochondria, and plasma membrane through the cryopreservation process (prefrozen semen and thawed semen) using EG as a cryoprotectant agent to different inclusion percentages (6, 8, and 10%).

## 2. Materials and Methods

### 2.1. Animals and Semen Collection Semen

Three-year-old *S. cuspicaudus* breeders from the Fishculture Research Institute at the University of Córdoba (CINPIC, Montería, Colombia, 8°47.5′ N and 75°51.8′ W) were kept in 400 m^2^ in earth ponds at a density of 0.5 kg/m^2^. The fish were fed twice daily using a commercial diet containing 45% net protein and supplemented with forage fish (*Oreochromis niloticus* juveniles) once a week.

Selected males (n = 18) in the spermiation phase at the reproductive mid-season (July-August) had a total length of 47.9 ± 2.2 cm and weighed 416.9 ± 123.8 g; for females (n = 4) in the final maturation phase, a length of 47.5 ± 29.4 cm and a weight of 592.2 ± 258.7 g was recorded. Selected breeders were transferred to circular-shaped, 3 m^3^ useful volume tanks with steady water flow (5 L/min). The fishes were kept for 48 h to acclimate them to the experimental conditions and reduce the stress from handling and environmental changes.

Selected males were treated with a single dose of salmon GnRH analog (12 µg/kg of live weight) applied to the pectoral fin base. Twelve hours later, semen was collected into 2.0 mL Eppendorf vials by hand stripping. The urogenital papilla was dried using a blotting towel, and the initial ejaculate was discarded to avoid contamination with water, urine, and feces.

### 2.2. Concentration and Sperm Kinetics

For the motility duration, 0.25 µL of fresh semen was placed onto a Makler chamber (Sefi Medical, Israel) and was activated with 75 µL of distilled water (1:300 dilution). The duration time was measured from activated until movement stopped in the spermatozoa [33]. Sperm concentration was assessed using 1 µL of semen mixed with 699 µL of 6% glucose (1:700 dilution); then, an aliquot (10 µL) of this dilution was placed in a Makler chamber for analysis. Sperm concentration was calculated using the computer-assisted sperm analysis software (CASA) Sperm Class Analyzer (Microptic, SCA^®^, Barcelona, Spain).

Analysis of sperm kinetics was performed on fresh, prefrozen (fresh semen-diluted), and thawed semen. The activation of spermatozoa was carried out by distilled water (1:300 dilution), and the parameters total motility and motility types (rapid, medium, slow) were recorded by the CASA software (Microptic, SCA^®^, Barcelona, Spain) linked to a camera of high speed (50 frames/s). Sperm were analyzed no later than five seconds after motility activation. The percentage of rapid sperm (>100 µm/s), medium sperm (45 to 100 µm/s), slow sperm (10 to 45 µm/s) sperm velocities, as well as that non-motile sperm (static) were obtained. The curvilinear (VCL) and linear (VSL) velocities were also estimated. Duration of motility, sperm concentration, total motility, and sperm velocity was run in triplicate to obtain the average from each semen sample analyzed.

### 2.3. Semen Cryopreservation, Freezing, and Thawing

Ethylene glycol (EG) (Sigma, St. Louis, MO, USA) was used as a cryoprotectant agent (CPA) at three inclusion percentages (6, 8, and 10%) combined with 6% glucose and 5% skim milk powder. The semen was diluted at a ratio of 1:4 (semen: extender, 27 ± 1 °C). A 1 mL aliquot was taken from this dilution after five minutes of exposure to cryoprotective solutions (pre-frozen semen) to assess damages and sperm kinetics. In all cases, the semen used in the trials was obtained from a mixture of six males (pool) with total motility greater than 90%. Motility and sperm velocity, damage to plasma membrane (Mem-d) and mitochondria (Mit-d), and DNA fragmentation (DNA-f) were evaluated for prefrozen and thawed semen. The damage was also analyzed in fresh semen (control) to identify possible effects of exposure to cryoprotectant, cryoprotectant inclusion level, and the freezing–thawing process.

Diluted semen was packaged in 2.5 mL cryotubes (Minitüb, Tiefenbach, Germany) and frozen in nitrogen vapors into a dry shipping container of 4 L (MVE, USA) for 30 min. The cooling rate was 27.3 °C/min from 28 to −20 °C, 29.9 °C/min from −20 to −100 °C, and 5.5 °C/min from −100 to −196 °C [34]. Frozen cryotubes were transferred to a 34 L cryo container (MVE, St Paul, MN, USA) and dipped directly into liquid nitrogen. Frozen cryotubes were thawed by direct dipping into a serological water bath (Memmert, WNB 7-45, Schwabach, Germany) at 35 °C for 90 s.

The osmolarity of the seminal plasma and the cryoprotective solutions at the different EG inclusion rates (6, 8, and 10%) was measured with an osmometer (Precision Systems, Osmette III, Pleasanton, CA, USA).

### 2.4. Damages to Sperm Membrane (Mem-d), Mitochondria (Mit-d), and DNA Fragmentation (DNA-f)

Mem-d and Mit-d were assessed in fresh, prefrozen, and thawed semen in 1, 10, and 10 µL aliquots, respectively. Sample staining was performed by adding 1 mL of a solution containing 3,3′-dihexyloxacarbocyanine iodide (DiOC_6_(3)) (70 nM) and 2 µg/mL propidium iodide (PI) (Sigma, St. Louis, MO, USA), incubating in the dark for 20 min, and then analyzing the samples by flow cytometry. DiOC_6_(3) staining allowed us to distinguish cells showing high DiOC_6_(3) uptake (viable cells) and low uptake (mitochondrial damage with no sperm membrane damage). Propidium iodide staining showed sperm membrane alterations. A FACSCanto II flow cytometer (BD Biosciences, San José, CA, USA) equipped with a 488 nm wavelength laser for excitation, 530/30 nm wavelength detectors for DIOC_6_(3) fluorescence detection, and 670 nm for PI were used.

DNA fragmentation was tested in fresh, prefrozen (fresh semen-diluted), and thawed sperm taking 10, 100, and 100 µL aliquots, respectively. Samples were fixed using 3 mL of 70% ethanol (Sigma, St. Louis, MO, USA). Subsequently, they were incubated for 12 h at 20 °C, washed using calcium-free, ultrafiltered BD PBS, vortexed, and centrifuged at 2500 rpm for 10 min at 4 °C to separate semen from the cryoprotective solution. The supernatant was discarded, and the pellet was resuspended in 300 µL of propidium iodide (PI) + RNase A (20 U) for every million cells. The samples were then vortexed and incubated in the dark for 20 min at room temperature. The samples were then analyzed by flow cytometry. To discriminate sperm cell (haploid) from non-sperm cell events (diploid), chicken red blood cells and ram T-cells from the DNA QC commercially available kit (BD Biosciences, USA) was used as system linearity controls and for the coefficient of variation. Samples were analyzed by a flow cytometer (BD Biosciences, FACS CANTO II, San José, CA, USA) with a 488 nm solid-phase excitation laser and fluorescence detection at 585/42 nm.

In fresh semen samples, membrane (Mem-d) and mitochondria (Mit-d) damage, as well as DNA fragmentation (DNA-f), were assessed fifteen minutes after collection. In prefrozen sperm, parameters were evaluated five minutes after diluting the semen into the extender. For thawed semen, the evaluation was performed immediately after thawing. The results show the spermatozoa percentage exhibiting mitochondrial and membrane damage and haploid cells with fragmented DNA.

### 2.5. Fertilization and Hatching Rates

Fertilization and hatching rates were estimated using 1.0 g of oocytes (~1104 oocytes/g) obtained by the induction of females in the final maturation phase using a single dose of salmon GnRH analog (12 µg/kg of live weight). The oocytes were taken from a spawning mixture of two females (pool). Fertilization was performed using 400 μL (~4.1 × 10^6^ spz/µL) of thawed semen from each treatment, activated using 10 mL of distilled water in 20 mL vials. Incubation was performed in 2 L-capacity experimental incubators with a 0.05 L/min upflow. Fresh semen was used as the control. At five-hour post-fertilization (hpf), when eggs were at the final phase of gastrulation (blastopore closure), a fertilization rate assessment from three aliquots (n = 80–100 eggs) of every incubator was performed. Eggs were placed in Petri dishes, and the viable embryo count was determined using an optical stereoscope (Leica, Wetzlar, Germany) and image analysis software (Zeiss, Axiovision 4.7, Oberkochen, Germany). The appearance of viable embryos was translucent or hyaline, with no detachment of cell material and a normal aspect. On the contrary, non-viable embryos were opaque or whitish [4]. Counting was performed three times, and the average value for each incubator was calculated. The fertility rate was estimated by using the equation below.

Fertilization = (N° of viable embryos/N° of analyzed embryos) × 100(1)

At 11 hpf, the hatching rate of embryos in the final faringulation phase (free-tailed embryo) was estimated, taking three 80-to-100 embryo aliquots and counting the viable embryos (the translucent, motile ones) and non-viable (the opaque and whitish ones). The same equation to estimate the fertilization rate was used. The fertility and hatching test was performed twice. In each trial, the treatments were evaluated in triplicate.

Recorded values for dissolved oxygen (7.1 ± 0.7 mg/L), temperature (26.3 ± 0.2 °C), pH (7.5 ± 0.1), hardness (52.7 ± 4.9 mg CaCO_3_/L), alkalinity (69.8 ± 8.8 mg CaCO_3_/L), and total ammonia (0.01 ± 0.004 mg/L) from water used for the incubation of eggs fertilized with fresh and thawed semen were within the range usually set for incubating Neotropical fish, such as *S. cuspicaudus* [35].

### 2.6. Statistical Analysis

All variables are presented as the mean ± SD, and previous to analysis, percentage data were standardized by square root transformation. All variables were subjected to one-way analysis of variance (one-way ANOVA) test and then followed by Tukey’s multiple range test (*p* > 0.05) to determine the best treatment. The data were analyzed with the software package SAS ver. 9.1.

## 3. Results

### 3.1. Seminal Plasma Osmolarity, Concentration, and Sperm Kinetics

Fresh semen of *S. cuspicaudus* (n = 18) recorded semen volume of 1.6 ± 0.4 mL, sperm concentration of 20,403.3 ± 6740 × 10^6^ spz/mL, and motility duration of 44.3 ± 6.3 s. The seminal plasma osmolarity was 273.3 ± 7.2 mOsmol/kg, while the cryoprotective solutions were 1905 mOsmol/kg for 6% EG and greater than 2000 mOsmol/kg for 8 and 10% EG (equipment measurement limit).

The total motility, motility types (rapid, medium, slow and static), and sperm velocity (VCL, VSL) in fresh, prefrozen, and thawed sperm are shown in Table 1. The total motility for fresh sperm was 95.1 ± 2.7%, showing a significant difference between prefrozen and thawed sperm (*p* < 0.05). The total motility in prefrozen sperm decreased from 61 ± 15.3% (6% EG) to 33.8 ± 11.6% (10% EG) (*p* < 0.05). In thawed sperm, total motility ranged between 35 ± 5.7% (6% EG) and 31.9 ± 2.1% (8% EG), showing no significant difference between these values, even with prefrozen semen to 10% EG (*p* > 0.05).

In fresh semen, the percentages of rapid (18.6 ± 5.9%) and medium motility sperm (58.0 ± 9.8%) showed a significant difference compared with prefrozen and thawed semen (*p* < 0.05). In prefrozen semen, rapid motility ranged between 0.9 ± 1.2% (6% EG) and 0.2 ± 0.2% (10% EG) (*p* > 0.05); in thawed semen, no rapid motility was recorded sperm. Medium motility sperm in prefrozen semen decreased from 16.9 ± 7.2% (6% EG) to 1.7 ± 0.2% (10% EG), showing the difference between these two values (*p* < 0.05). In thawed semen, medium motility ranged from 1.9 ± 0.6% (6% EG) and 1.3 ± 0.7% (8% EG), and no difference between these values was found, even with prefrozen semen to 10% EG (*p* > 0.05).

In fresh semen, the percentages of slow motility sperm (20.5 ± 5.3%) and static sperm (5.5 ± 3.3%) showed a significant difference with prefrozen and thawed semen (*p* > 0.05). In prefrozen semen, slow motility increased (30.4 ± 9.4%–43.3 ± 9.6%), and similar values were observed in thawed semen (30.6 ± 1.6%–33.1 ± 5.2%); no significant difference was observed for slow motility between prefrozen and thawed semen to the different ethylene glycol inclusion percentages (*p* > 0.05). Static sperm in prefrozen semen increment from 39 ± 15.4% (6% EG) to 66.2 ± 11.6% (10% EG) (*p* < 0.05). The amount of non-motile sperm (65 ± 5.7%–68.1 ± 2.1%) in thawed semen did not show a significant difference in the different ethylene glycol inclusion percentages, even with prefrozen semen to 10% EG (*p* > 0.05).

The fresh semen VCL (72.9 ± 5.5 µm/s) showed a significant difference with prefrozen and thawed semen (*p* < 0.05). The prefrozen semen VCL decreased from 34.5 ± 4.2 µm/s (6% EG) to 23.3 ± 3.2 µm/s (10% EG) (*p* < 0.05); whereas the thawed semen VCL ranged between 23.2 ± 2.6 µm/s (6% EG) and 21.1 ± 1.3 µm/s (10% EG), no significant difference was observed between these values, even with prefrozen semen to 10% EG (*p* > 0.05). The fresh semen VSL (55.9 ± 8.8 µm/s) showed a significant difference with prefrozen and thawed semen. In prefrozen semen, VSL decreased from 18.5 ± 5.8 µm/s (6% EG) to 7.7 ± 3.6 µm/s (10% EG) (*p* < 0.05), whereas in thawed semen, VSL ranged between 7.1 ± 1.2 µm/s (10% EG) and 6.9 ± 1.1 µm/s (8% EG), but no significant difference was observed for these values, even with 10% EG prefrozen semen (*p* > 0.05).

### 3.2. Damages in Prefrozen and Thawed Sperm

Membrane damage (Mem-d), mitochondrial damage (Mit-d) and DNA fragmentation (DNA-f) in fresh, prefrozen, and thawed sperm are shown in Table 2. Mem-d results in fresh semen were 1.0 ± 0.5%, with no difference observed from prefrozen semen when 6% EG (2.4 ± 1.1%) and 8% EG (3.0 ± 2%) were used. The highest values for Mem-d were recorded in thawed semen, which ranged between 24.5 ± 2.2% (8% EG) and 26.6 ± 3.4% (10% EG), with no difference observed between these values (*p* > 0.05), although there was a difference with the values obtained in the prefrozen semen (*p* < 0.05).

In fresh semen, lower percentages of Mit-d were recorded (1.4 ± 0.9%), showing a significant difference with prefrozen and thawed semen (*p* < 0.05). Mit-d of prefrozen semen increased concerning fresh semen, with values ranging between 16.1 ± 2% (6% EG) to 18.3 ± 5.7% (10% EG), with no showing significant difference for these values (*p* > 0.05). For thawed semen, however, Mit-d results continued to increase to values between 47.8 ± 5.4% (10% EG) and 51.4 ± 2.7% (8% EG), but no significant difference was observed for these values (*p* > 0.05). Nonetheless, there was a significant difference in Mit-d values compared to those recorded in prefrozen semen (*p* < 0.05).

In fresh semen, lower percentages of DNA-f were recorded (2.4 ± 0.8%), with a significant difference with prefrozen and thawed semen (*p* < 0.05). DNA-f for prefrozen semen ranged between 6.7 ± 2.5% (6% EG) and 9.5 ± 2.6% (10% EG), without a significant difference for these values (*p* > 0.05). In thawed semen, however, DNA-f values increased from 37.9 ± 2.8% (8% EG) to 44.4 ± 3.4% (6% EG), but no statistically significant difference for these values was shown (*p* > 0.05). However, there was a significant difference in prefrozen semen (*p* < 0.05).

### 3.3. Fertilization and Hatching Rates

**Figure 1:**
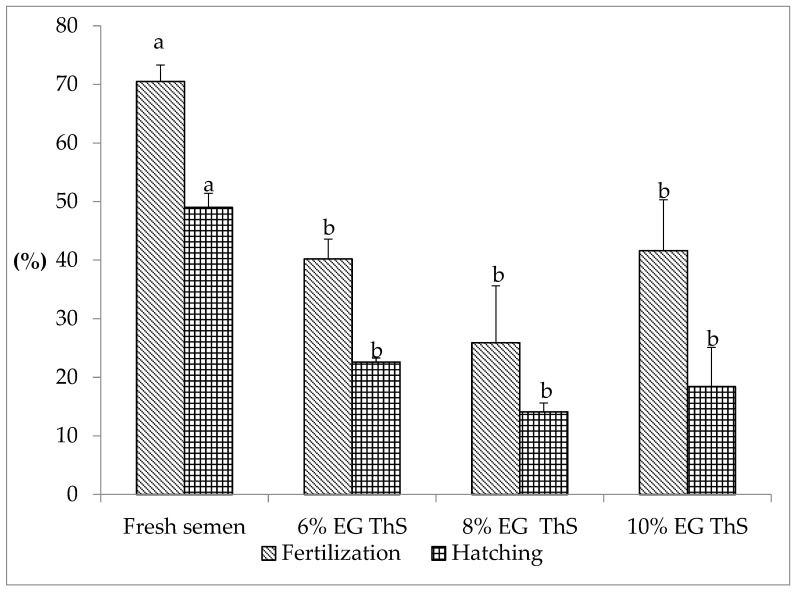
Fertilization and hatching rates of *S. cuspicaudus* using fresh or cryopreserved semen with different inclusion percentages (6, 8, and 10%) of ethylene glycol (EG). Different letters between treatments indicate differences (*p* < 0.05). ThS, thawed sperm.

The fertilization and hatching rates of *S. cuspicaudus* using fresh semen and thawed semen are shown in Figure 1. Fresh semen registered a fertilization rate of 70.5 ± 2.8% and a hatching rate of 49 ± 2.4%, showing a significant difference with thawed semen (*p* < 0.05). The fertilization rate using thawed semen ranged from 41.6 ± 8.7% (10% EG) to 25.9 ± 9.7% (8% EG), and no statistically significant difference was observed for these values (*p* > 0.05). Likewise, the hatching rate varied from 22.6 ± 0.7% (6% EG) to 14.1 ± 1.5% (8% EG), with no statistically significant difference observed for these values (*p* > 0.05).

## 4. Discussion

*S. cuspicaudus* fresh semen used in this study was collected at reproductive mid-season (July–August). Previous studies suggest an adequate semen quality of *S. cuspicaudus* when it shows total motility greater than 80%, duration of motility in the range between 36 and 46 s and sperm concentration greater than 14.000 million spz/mL [3,4]. The semen used in the present study registered values in these ranges. Furthermore, fresh sperm was characterized by high total motility (>95%) and low rates of Mem-d (<1.0%), Mit-d (<1.4%), and DNA-f (<2.4%), which suggests that the semen was of good quality.

Total motility, motility types (rapid, medium, and slow), and sperm velocity (VCL, VSL) in prefrozen semen (fresh semen-diluted) were lower compared to fresh semen (*p* < 0.05), showing that total motility and sperm velocities decrease as the EG inclusion percentage increases. Lower values for these parameters were achieved when 10% EG was used; however, rapid, medium, and low motility were affected, regardless of the EG inclusion percentage used. In thawed semen, total motility, types of motilities (rapid, medium, and slow), and velocities (VCL, VSL) indicate that the sperm was affected by freezing and thawing processes. After thawing, no rapid spermatozoa were registered, the percentage of medium motility sperm decreased, slow and immotile sperm increased, and sperm velocity slowed (VCL, VSL).

The results in prefrozen semen suggest that the effects on sperm motility, motility types, and velocity are caused by hyperosmotic shock and cryoprotectant agent toxicity. The cryoprotective solution used in this study was highly hyperosmotic (6% EG = 1905 mOsmol/kg; 8 and 10% EG > 2000 mOsmol/kg). Cuevas-Uribe et al. [36] attributed the decreased motility to the high osmotic pressures caused by high concentrations of cryoprotectants and toxic effects. In thawed semen, the thermal shock caused by freezing and thawing is responsible mainly for the damage caused.

An increase in Mem-d, Mit-d, and DNA-f was observed in prefrozen semen compared with fresh sperm(*p* < 0.05), which shows the harmful effects on semen quality during exposure to CPA. Of all structures assessed, mitochondria were the cell organelle most affected at the prefrozen phase. In this study, the equilibration time was five minutes, and during this time, Mit-d increased 11- to 13-fold compared to fresh semen; however, the Mit-d did not show significant differences (*p* < 0.05) in the different EG inclusion percentages in the assessed range (6 to 10%). On the other hand, the membrane was the less affected structure (less than 5%) during the prefrozen phase, increasing from 2.4 to 4-fold regarding fresh semen, although the membrane underwent greater damage when 10% EG was included compared to fresh semen (*p* < 0.05). Fragmentation of DNA in prefrozen semen increased 2.8- to 4-fold compared to fresh semen, regardless of the EG inclusion percentage (6 to 10%). The results suggest that of the cell structures analyzed, mitochondria are more sensitive to damage caused by CPA toxicity and osmotic shock in the prefrozen phase.

In thawed semen, Mem-d ranged between 24.5 and 26.6%, regardless of the EG inclusion percentage rate, with a 6- to 11-fold increase compared to prefrozen semen and 24.5–26.6-fold to fresh semen. It has been suggested that changes in the plasma membrane during cryopreservation are strongly associated with the CPA used and inclusion percentage and diluent, which would cause cell apoptosis. Keeping the plasma membrane intact is critical to the fertilizing capacity of fish sperm; it can fuse with the oocyte by fluidity, fusogenicity, and permeability [18]. Cabrita et al. [37] observed that membrane sensitivity leads to changes in stability and increased membrane fragility during the freezing and thawing process, resulting in increased permeability to water and cations to osmotic stress. Damage to the plasma membrane causes a loss of intracellular components such as proteins, enzymes, and other components, coeluting to reduce metabolic activity and, consequently, decrease sperm quality [12]. Another work has found alterations in several proteins involved in multiple functions associated with metabolism, as well as the stress response, signal transduction, apoptosis, transcription, translation, protein folding, and DNA repair [13]. These protein alterations compromise physiologic functions such as motility, fertilizing capacity, and early events after fertilization. Injury to the sperm membrane upon cryopreservation may be due to osmotically-induced cryoprotectant toxicity or insufficient concentration of cryoprotectant in the cell interior, allowing the formation of intracellular ice crystals [15].

In thawed semen, damage to mitochondria increased 2.6- to 3-fold relative to prefrozen semen and 34- to 37-fold compared to fresh semen, indicating that mitochondria also suffer significant injury due to freezing and thawing. In thawed semen, there was no significant difference (*p* < 0.05) between the damage of the mitochondria and the EG inclusion percentage tested (6, 8, 10%). Oxidative stress is considered a major cause of mitochondrial damage because it promotes the release of reactive oxygen species (ROS), which affects mitochondrial respiration efficiency [22,38].

In thawed semen, DNA-f increased 3.3- to 6.6-fold concerning prefrozen sperm and 13- to 18.5-fold regarding fresh semen. These results suggest that DNA-f was also affected at the freezing–thawing process, but without statistical differences between the different EG inclusion percentages analyzed (6, 8, 10%). DNA fragmentation affects embryo development and increases early-stage embryo mortality [39,40,41]. It has been suggested that nuclear DNA undergoes fragmentation due to the production of ROS [5], so oxidative stress modifies nuclear DNA histones and may affect gene expression [42,43]. Nitrogenated DNA bases (particularly guanine) are the main target of ROS, generating 8-hydroxy,2′-deoxyguanosine (8OHdG) [5]. This reaction weakens the bond between guanine and the adjacent ribose unit, causing the oxidized base’s loss, thus destabilizing the DNA structure and resulting in the fragmentation of localized strands [44] found that DNA damage in cryopreserved sperm of rainbow trout was negatively related to fertilization; however, the low fertilizing capacity was not entirely attributed to DNA alteration. It has been suggested that chromatin damage has a much more critical effect on early embryo development rather than in fertilization, under its role in controlling gene expression at the early stages of embryo development [45,46,47,48].

While the ethylene glycol inclusion percentage tested (6 to 10%) affected semen quality both at the pre-freezing and thawing phase, no significant differences were observed regarding the fertilizing capacity of thawed semen (*p* > 0.05), so it might be suggested that EG rate in the range of 6 to 10% shows similar fertilization rates if freezing and thawing conditions like those described in this study are used. Nevertheless, it has been found that an EG included above 10% affects the quality of *S. cuspicaudus* cryopreserved-thawed semen [4]. Moreover, in *Piaractus brachypomus*, it was reported that the EG rate of 10% or higher causes a decrease in semen quality, affecting fertilizing capacity [49]. In addition, in the cryopreservation of *Brycon amazonicus* semen with EG above 10%, the quality of the thawed semen was affected [34].

The decrease in the fertility and hatching rates of thawed semen regarding fresh semen is suggested because of the injuries that occurred both in the pre-freezing and the freezing/thawing processes. In the present study, it was established that semen exposure to cryoprotective solutions and the freezing and thawing process caused DNA fragmentation and damage to mitochondria and the plasma membrane. The DNA, mitochondria, and membrane integrity are affected in each phase of the cryopreservation process, and these structures are essential for the success of embryonic development. The decrease in the percentage of viable cells and the increase in DNA fragmentation causes a reduction in the fertilization capacity of spermatozoa and increases the probability that an egg will be fertilized by a spermatozoon with damaged DNA, increasing non-viable embryos [50].

This study’s results show that plasma membrane and mitochondria damage and DNA fragmentation suffered by the sperm during the cryopreservation process (pre-freezing, freezing, and thawing) affect its fertilizing capacity. Although sperm DNA-f may exhibit motion and have fertilizing capacity, the embryo would not be viable. However, according to Kopeika et al. [25], sperm cells may possess features that prevent damage and develop cryo-resistance during freezing.

Fertilization is achieved, although the percentage of non-motile spermatozoa in thawed sperm is high. This is possible through sperm–oocyte interactions and a micropylar apparatus in fish oocytes allowing non-motile sperm in the presence of vibration to penetrate the ooplasma, producing oocyte fertilization [51,52]. This phenomenon has been attributed to oocyte-related, but unclear factors, which might activate non-motile sperm, as the presence of oocyte-released agents in the form of small polypeptide molecules synthesized in cell follicles and accumulated in the corium (motility initiation factors) can cause sperm motility hyperactivity in some fish species [53,54].

In conclusion, the present study demonstrated that the mitochondria, plasma membrane, and DNA integrity suffer considerable damage both in pre-frozen and in freezing/thawed semen when it is cryopreserved with EG in inclusions between 6 and 10% that affect its fertilizing capacity, which is reduced to half of that obtained with fresh semen. In the pre-freezing phase, the Mit-d and DNA-f are independent of the inclusion level (6 to 10%) when the equilibration time is five minutes, but Mem-d is higher when EG is included at 10% in this phase. In freezing–thawing, the cryoprotectant inclusion percentage (6 to 10%) is independent of the damage it causes to membranes, mitochondria, and DNA; therefore, it is suggested that a cryoprotective solution composed of 6% EG, 6% glucose, and 5% skimmed milk powder is a useful protocol for cryopreservation of *S. cuspicaudus* semen. Further studies are required to optimize semen cryopreservation methods and examine the injuries that semen suffers in this process, particularly long-term effects, such as larval viability, to increase cryopreservation success and for their potential use in conservation programs.

## Figures and Tables

**Table 1:** Total motility (tM), motility types (rapid, medium, slow, static), and sperm velocities in fresh, prefrozen (fresh sperm diluted), and frozen-thawed sperm from *S. cuspicaudus* with different inclusion percentages (6, 8, and 10%) of ethylene glycol (EG). Different letters indicate differences (*p* < 0.05). VCL, curvilinear velocity; VSL, linear sperm velocity.

Parameters	Fresh Sperm	Prefrozen Sperm			Thawed Sperm		
		6% EG	8% EG	10% EG	6% EG	8% EG	10% EG
tM (%)	95.1 ± 2.7 ^a^	61.0 ± 15.3 ^b^	51.2 ± 10.5 ^b,c^	33.8 ± 11.6 ^d^	35.0 ± 5.7 ^c,d^	31.9 ± 2.1 ^d^	32.5 ± 5 ^d^
Rapid (%)	18.6 ± 5.9 ^a^	0.9 ± 1.2 ^b^	0.3 ± 0.3 ^b^	0.2 ± 0.2 ^b^	0.0 ± 0.0	0.0 ± 0.0	0.0 ± 0.0
Medium (%)	58.0 ± 9.9 ^a^	16.9 ± 7.2 ^b^	9.9 ± 6.5 ^b^	1.7 ± 0.2 ^c^	1.9 ± 0.6 ^c^	1.3 ± 0.7 ^c^	1.5 ± 0.5 ^c^
Slow (%)	20.5 ± 5.3 ^b^	43.3 ± 9.6 ^a^	41.0 ± 5.1 ^a^	30.4 ± 9.4 ^a,b^	33.1 ± 5.2 ^a^	30.6 ± 1.6 ^a b^	31.1 ± 4.7 ^a b^
Static (%)	5.5 ± 1.9 ^d^	39.0 ± 15.4 ^c^	48.8 ± 10.5 ^bc^	66.2 ± 11.6 ^a b^	65.0 ± 5.7 ^a,b^	68.1 ± 2.1 ^a^	67.9 ± 5.1 ^a,b^
VCL (µm/s)	72.9 ± 5.5 ^a^	34.5 ± 4.2 ^b^	28.2 ± 5.1 ^b,c^	23.3 ± 3.2 ^c,d^	23.2 ± 2.6 ^c,d^	21.1 ± 1.8 ^d^	21.1 ± 1.3 ^d^
VSL (µm/s)	55.9 ± 8.8 ^a^	18.5 ± 5.8 ^b^	13.4 ± 4.7 ^b,c^	7.7 ± 3.6 ^c^	7.0 ± 0.6 ^c^	6.9 ± 1.1 ^c^	7.1 ± 1.2 ^c^

**Table 2:** Damages in prefrozen and frozen-thawed *S. cuspicaudus* sperm with different inclusion percentages (6, 8, and 10%) of ethylene glycol (EG). Different letters indicate differences (*p* < 0.05). Mem-d, membrane damage; Mit-d, mitochondrial damage; DNA-f, DNA fragmentation.

Damage	Fresh Semen	Prefrozen Semen			Thawed Semen		
		6% EG	8% EG	10% EG	6% EG	8% EG	10% EG
Mem-d (%)	1.0 ± 0.5 ^c^	2.4 ± 1.1 ^bc^	3.0 ± 2.0 ^b,c^	4.1 ± 1.1 ^b^	24.8 ± 2.2 ^a^	24.5 ± 2.2 ^a^	26.6 ± 5.4 ^a^
Mit-d (%)	1.4 ± 0.9 ^c^	16.1 ± 2 ^b^	16.5 ± 3.4 ^b^	18.3 ± 5.7 ^b^	49.0 ± 7.3 ^a^	51.4 ± 2.7 ^a^	47.8 ± 5.4 ^a^
DNA-f (%)	2.4 ± 0.8 ^c^	6.7 ± 2.5 ^b^	6.8 ± 3.3 ^b^	9.5 ± 2.2 ^b^	44.4 ± 3.4 ^a^	37.9 ± 2.8 ^a^	38.6 ± 6.8 ^a^

## Data Availability

All data provided in this manuscript were appropriately cited in the tables, figures, and reference section.
